# Dietary Diversity of Women across Agricultural Seasons in the Kapchorwa District, Uganda: Results from a Cohort Study

**DOI:** 10.3390/foods11030344

**Published:** 2022-01-26

**Authors:** Irmgard Jordan, Anna Röhlig, Maria Gracia Glas, Lydiah Maruti Waswa, Johnny Mugisha, Michael B. Krawinkel, Ernst-August Nuppenau

**Affiliations:** 1 Center for International Development and Environmental Research, Justus Liebig University Giessen, 35390 Giessen, Germany; aroehlig@gmx.de (A.R.); maria.g.glas@zeu.uni-giessen.de (M.G.G.); 2 Department of Human Nutrition, Egerton University, Nakuru P.O. Box 536-20115, Kenya; lwaswa@egerton.ac.ke; 3 School of Agricultural Sciences, Makerere University, Kampala P.O. Box 7062-10218, Uganda; johnny.mugisha@mak.ac.ug; 4 Institute of Nutritional Sciences, Justus Liebig University Giessen, 35392 Giessen, Germany; krawinkel@fb09.uni-giessen.de; 5 Agricultural and Environmental Policy, Justus Liebig University Giessen, 35390 Giessen, Germany

**Keywords:** agricultural seasons, dietary diversity, market linkages, farm households, women, Uganda

## Abstract

Small-holder farm households in developing countries mainly depend on rain-fed agriculture activities, thus seasonality affects eating habits and contributes to micronutrient deficiencies. This study assessed women’s dietary diversity score (WDDS) across three agricultural seasons in the Kapchorwa District, Uganda. In each season, 445 women with under five-year-old children from small-holder farm households were interviewed on socio-demographic characteristics as well as dietary practices between May 2016 and January 2017. Linear regression models estimated differences in WDDS across seasons. The mean WDDS at the lean, harvest and post-harvest seasons was 4.33 ± 1.21, 4.63 ± 1.30 and 4.36 ± 1.21, respectively. Mean WDDS was higher in urban regions across all seasons. Women in urban regions generated more income through off-farm activities, had better access to markets and consumed significantly more ‘meat, poultry and fish’ (*p* < 0.001), dairy products (*p* < 0.05), ‘vitamin A-rich fruits and vegetables’ (*p* < 0.001) and ‘other vegetables’ (*p* < 0.001) during the lean season, whilst rural women ate more dark green leafy vegetables (*p* < 0.001) during the same period. Poorer households in the rural setting were more likely to be affected by seasonally limited food availability and accessibility. Hence, strengthening the linkages between market participation, agricultural activities, improved food storage and preservation techniques and dietary intake is recommended.

## 1. Introduction

The overall change in climate has influenced seasons making food availability and access even more unpredictable. Small-holder farmers who depend mainly on rain-fed agricultural activities experience poor harvests with fluctuating weather conditions and thus suffer from reduced food availability and household incomes, higher food prices and consequently, reduced food consumption [1,2,3,4]. Seasonal changes in food availability and access have been shown to affect dietary diversity and subsequently, nutrient adequacy of diets consumed by women from small-holder farming households in Sub-Saharan Africa [5,6,7,8]. The consequences of this inadequate nutrient intake among women of reproductive age (15–49 years old), who have increased nutritional requirements, are increased burdens of micronutrient deficiencies, increased incidences and severity of infections, poor pregnancy outcomes and even mortality.

In Uganda, the national prevalence of anaemia among women aged 15–49 years old was 32% in 2016 and even much higher among children below five years (53%) [9]. Whereas 9% of the under-five-year-old children were estimated to be vitamin A deficient with a higher prevalence among stunted children (35%) in 2016 [9,10]; no recent national data were found for vitamin A deficiency among women. Still, these figures indicate inadequate dietary intake in the respective families, and measures to increase availability and access to more diverse diets have been recommended to enhance the nutrition and health status of small-holder farmers and their families, in particular among women of reproductive age (15–49 years) [11,12].

A commonly used indicator to measure the diet quality for women is the minimum dietary diversity score (MDD-W) [13,14]. Women who consume foods from at least five out of ten defined food groups are more likely to achieve adequate micronutrient intake [13]. Several studies have demonstrated the association between consumption of diverse diets and attainment of nutrient adequacy among adults and particularly among women of reproductive age [6,15,16,17]. However, dietary patterns and portion sizes of food items depend on various aspects, such as land availability, market access, literacy level, outside temperature, rainfall patterns and the wealth of a household [18,19]. Moreover, it is also known that seasonality contributes to variations in dietary patterns [6,8]. Fluctuations in dietary diversity and nutritional status among women during the lean period and shortly before the beginning of the harvest seasons have been reported, for example, from Burkina Faso [3]. Hence, households depending on rain-fed agricultural production are more likely to be affected by seasonal food shortages.

A study conducted in Uganda revealed that the dry season was especially associated with increased food insecurity, i.e., with a majority of households experiencing greater difficulties in acquiring sufficient quantities and quality of food [20]. The same study indicated that the effect of seasonal variation on food security is modified by employment, wealth and community location [20]. In yet another study conducted in Uganda, household dietary diversity was found to vary significantly with agricultural seasons and livelihood systems between households within pastoral and agro-pastoral livelihood systems [21]. However, these studies looked at livelihoods and indigenous populations which are very different from the population in Eastern Uganda in the Mt. Elgon Region. The climate conditions at Mt. Elgon, our research area, foster rain-fed coffee-, banana- and vegetable-cash crop production systems which are linked to national and international markets. Still, malnutrition rates are high in this region, far above the national average, and of public health concern (36% stunting vs. 29% at the national level) [9]. The information on seasonal food consumption and dietary diversity for women in Eastern Uganda is very limited. In particular, no information is available with regards to the differences in diets between those from rural areas or rural suburbs surrounding a district town such as Kapchorwa. Poorly diversified farming systems, small plots (land areas), soil degradation and low dietary diversity are considered the main reasons for the high prevalence of malnutrition among farm families in this district [22,23,24,25,26,27,28]. The male farmers in the region focus on cash crop production (mainly coffee, matoke, maize, Irish potatoes and cabbage) whilst women are mainly responsible for the subsistence food production [25].

The types of meals consumed in households in Kapchorwa District in Uganda consist mainly of starchy staples, such as bananas, roots, tubers and maize (posho), which are complemented with pulses and/or some seasonal dark green leafy vegetables. These diets are often poor in diversity. They provide especially insufficient amounts of micronutrients and animal source proteins [11,29].

This study was part of the larger project HealthyLAND [30] which aimed to discover linkages between diversified agricultural and dietary diversity among the small-holder farm households in the Kapchorwa District, as well as to identify and develop innovations for sustainable food and nutrition security. The specific objective of this study was to determine the influence of seasonal variations on the dietary diversity of women in a rural and urban setting in the Kapchorwa District, Uganda. This included an assessment of socio-economic factors mediating the effect of seasonality on dietary diversity.

## 2. Materials and Methods

### 2.1. Study Setting

**Figure 1:**
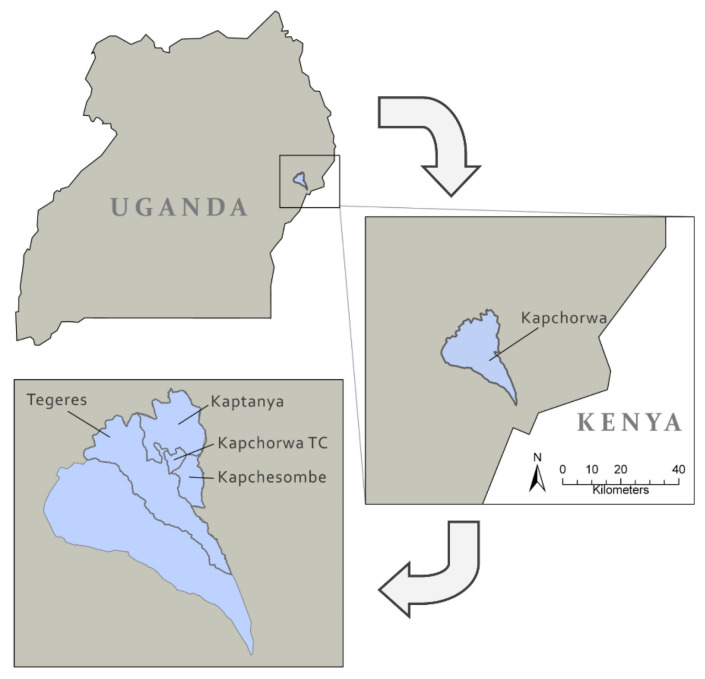
Location of the Kapchorwa District. Published under a CC BY license, with permission from Björn Weeser, original copyright 2019.

Four sub-counties in Kapchorwa District were included in the study and classified as “urban” or “rural” based on administrative boundaries and proximity to the urban centre of Kapchorwa town, the administrative centre of the District [31] (Figure 1).

Participants living in Kapchorwa Town Council (TC) represented the ‘urban’ subgroup whilst those from Kapchesombe, Kaptanya and Tegeres were classified as ‘rural’. The district covers four agro-ecological zones ranging from the lower to upper belt [22] allowing rain-fed agriculture, the main income source for its population. The rains usually allow two cropping seasons: a long season from March to July (Season A) and a shorter season from September to December (Season B) [22]. From September to December 2016, the rainfalls were lower than expected and the region suffered from a mild to severe drought in the respective period [32].

### 2.2. Study Design

A prospective cohort study design was applied during the study period. We started with an agriculture–nutrition baseline survey during the lean season in May/June 2016. Three and six months later, the baseline participants were followed up in two intermediate surveys to assess their dietary intakes during two other agricultural seasons representing the harvest season (late August/beginning of September 2016) and the post-harvest season (January, 2017). The term ‘post-harvest’ is associated with the period with very limited or no harvest activities left and most of the harvest from the “cash crops” has been sold. During this period, the farmers are usually left with cash which may or may not be used to purchase foods and/or have foods in stores as the land is dry and limited foods can still be harvested [25].

The sample size for the current study was the same as that of the overall HealthyLAND project which was calculated based on the women’s dietary diversity as the primary indicator. We used the formula for cluster randomised trials, as the overall project aimed to test intervention effects [33].

$$N=a*b*2*(((z(a÷2)+z(\beta ){)}^{2}\times (SD1^{2}+SD2^{2}))÷(M1-M2{)}^{2})\times (1+ICC\times (m-1))$$

*N* stands for total sample size, whilst *a* = the constant for drop out (1.12), and *b* = the constant to adjust for the unequal sample sizes in both groups (allocation ratio 1:2; *b* = 1.125)). *SD*1 and *SD*2 describe the expected standard deviation for the change in the women’s dietary diversity score in two different groups (1.5), and *M*1 and *M*2 are the respective mean values of the change (0 and 0.75, respectively). Each village was assumed to be a cluster unit. Alpha was set to 5% (without any adjustments for multiple comparisons) and power on 80%. To be able to adjust for intra-class correlation (*ICC*), a design effect (*DEFF*) was included and defined as: 𝐷𝐸𝐹𝐹 = 1 + 𝐼𝐶𝐶 (𝑚 − 1). Considering, that 11 households (m) per cluster would be measured, using a conservatively estimated *ICC* of 0.15, the *DEFF* was calculated = 1 + 0.15 × 11 = 2.5. The required sample size was n = 396 farm-households. As a result of the power calculation, the baseline study aimed at a minimum of 400 farm-households. Eligibility criteria to participate in the study included being a member of a farm-household with at least one child aged between 0 and 59 months, randomly selected from sampled villages identified by the agriculture research team.

During the first sampling stage, four sub-counties were purposively selected from the Kapchorwa District to cover all agro-ecological zones (uppermost, mid-upper, mid-lower and lowermost). Subsequently, 40 villages were selected proportional to population size (PPS) from the four sub-counties. During the baseline survey (May/June 2016; lean season), 11–12 households were randomly selected from each selected village, resulting in a total of 460 households. The same small-holder farm households were followed up during the harvest in August 2016 and post-harvest season (January 2017). In these follow-up surveys, we thus covered a total of three different agricultural seasons and focused on the dietary practices of the women.

The study was approved by the institutional review board of the Justus Liebig University, Giessen, Germany, and by the Review Board of the Makerere University, Kampala, Uganda (number 56/16).

### 2.3. Data Collection

The data for the study were collected using structured questionnaires through face-to-face interviews with the participants in their homesteads or farms by a team of 15 trained local enumerators with a minimum of an undergraduate degree in nutrition, agriculture or related fields. The enumerators were fluent in English and Kubsabiny, the local language spoken in the study area. Prior to the commencement of the baseline survey, the enumerators were intensively trained by a team comprising Ph.D. students under the supervision of senior scientists on data collection methods and administration of the tablet-based questionnaires through interviews with study participants. The questionnaires were programmed using the ODK software [34]. The primary caregiver of the children under five years were the main respondents during the interviews. The filled questionnaires were checked for completeness and any missing data and inconsistencies addressed at the end of each field day by the supervisory team to ensure quality data.

The structured questionnaire used at baseline included questions to assess both agriculture and nutrition practices of the participating small-holder farm households. The questions were selected from questionnaires that we had successfully applied in other studies already [5,35], and which we had adopted to the specific study environment. The questionnaire at baseline included questions that sought to collect information on the status of the small-holder farm household food security, dietary diversity, child feeding practices, health status, caregiver’s knowledge of child feeding practices and household dietary behaviours. Further data on crops grown, quantities harvested, income sources, as well as farming challenges, were collected. During the follow-up surveys, we focused on a 24 h dietary recall to calculate dietary diversity.

Refresher training was conducted for the enumerators who participated in the baseline survey and were re-hired to conduct the two follow-up surveys prior to commencement of each of the follow-up surveys during the harvest and post-harvest seasons. To ensure high respondent rates during the follow-up surveys, the participants from the sampled small-holder farm households were notified about the survey at least one day beforehand, and in case of absence, enumerators performed up to three follow-up visits. The objective of the study was explained to the participants prior to the interview and their informed written consent was obtained. Participation in the study was purely voluntary and participants were free to end the interviews and the study at any time.

### 2.4. Household Characteristics

The household questionnaire was used to gather information on the socio-demographic and economic characteristics of the study participants. For example, we asked for the age of household members, marital status, education level, main occupation, household size, type of housing, possession of valuable assets, such as furniture, mobile phone, bicycle, radio, television, car, truck and fridge, type of cooking facilities, access to and treatment of drinking water and availability of sanitation facilities, ownership of farm implements, land size and the number of different livestock species reared, including poultry, goats, sheep, cattle, donkeys and others. We also asked the participants to estimate the amount of time they spent to reach the nearest health facility and marketplace.

The information gathered on household possessions and livestock was used to construct a wealth index using principal component analysis [36,37]. This standard analysis is well accepted for urban but less so for rural areas as the rural poor might not be sufficiently differentiated [38]. We, therefore, followed Rutstein’s [36] recommendation to construct a composite wealth index to allow for comparisons between rural and urban households. This decision was also based on our observation that the livelihood of the study participants in Kapchorwa TC, which we had classified as urban, were very similar to those living in the more remote sub-counties. The wealth index was used to describe the socio-economic status of the study population with a high score indicating wealthier households and vice versa. For the computation of the index, the categories of included variables were transformed into binary variables, using 1 for improved and 0 for unimproved outcomes.

### 2.5. Dietary Diversity

The dietary diversity score of the women (mothers or primary caregivers of children aged below five years) was calculated based on data collected from single 24-h dietary recalls conducted at the individual level during the three seasons of the study. The selected women were asked by the trained enumerators to recall all the food items and drinks they had consumed the previous day from the time they woke up until they went to bed. A detailed description of all ingredients used in mixed dishes was recorded. No differentiation was made between weekdays and the weekend but feast days were intentionally not included. The data from the 24-h dietary recall was used to calculate the women’s dietary diversity score (WDDS) based on the 10 food group score recommended by FAO and FHI 360 [13], i.e., (i) grains, white tubers and plantain; (ii) pulses; (iii) nuts and seeds; (iv) dairy; (v) meat, poultry and fish; (vi) eggs; (vii) dark green leafy vegetables (DGLV); (viii) vitamin A-rich fruits and vegetables, (ix) other vegetables and (x) other fruits. A score of 1 was awarded when a food item was consumed from a particular food group, and 0 if none were consumed. Any woman who consumed foods from five or more out of the ten food groups during the previous day was considered to have achieved minimum dietary diversity for women (MDD-W), which is an indicator for assessing micronutrient adequacy among women of reproductive age [13].

### 2.6. Anthropometric Assessment

Anthropometric measurements of the women were taken by pairs of trained enumerators following standard procedures [39]. The body weight measurements were taken with the women wearing light clothing and no shoes standing upright on flat digital scales with a mother–child function (Seca 874, capacity: 200 kg). No uniform subtraction was done to acknowledge the weight of the clothing [40,41]. The measurements were taken twice and recorded to the nearest 0.1 kg. Height measurements were taken with the women barefoot and standing in an upright position using the stadiometer (Seca 213, measurement range: 20–205 cm). Weight and height were used to calculate the body mass index (BMI) for non-pregnant women.

### 2.7. Data Analysis

Completed interviews were exported from the tablets to an external hard drive, transformed and exported as *.csv files to the Statistical Package for Social Sciences (SPSS) software version 27.0 (IBM, 2020) for further analysis [42]. Stata’s (version 16) procedure-mixed was used for all regression models.

Descriptive analyses were performed in order to describe the socio-demographic and economic characteristics of the study participants for both rural and urban households and also across the three different seasons. To establish statistically significant differences for selected variables among both groups an independent *t*-test for normally distributed variables was used. The Mann–Whitney test was used for non-normally distributed variables, and nominal variables were tested with the Pearson chi-square test. Comparisons in food consumption patterns across different seasons within the same group were done using the Wilcoxon test.

To test whether differences in dietary diversity occurred due to the wealth of the household, a two-way ANOVA, including a posthoc analysis was performed. Moreover, Spearman’s rho correlation test was conducted to verify possible relations. The significance level was set at *p* < 0.05 for all tests.

Based on these findings, we conducted a regression analysis which was driven by the hypotheses that season, location and education mattered. In three models, the associations between the women’s dietary diversity as dependent variable and season and urban (Model 1), season, urban and wealth (index) (Model 2) and season, urban, wealth (index) and educational level of the mother of the child (Model 3) were analysed. Linear mixed models were used to account for the repeated measurements of the women’s dietary diversity. Random intercept models with household (=person) as cluster variable and independence of residuals fitted the data best.

All models are reported including the interaction season*urban. In Models 2 and 3, all interactions between the predictors were tested. The non-informative interactions were excluded for reasons of model parsimony, taking the relatively small sample size into account. Likewise, nonlinear associations between wealth and women’s dietary diversity, and between women’s educational level and dietary diversity, were tested but proved to be not informative.

## 3. Results

### 3.1. Socio-Demographic Characteristics of the Study Participants

The socio-demographic characteristics of the study participants from the rural and urban households during the three seasons are summarised in Table 1. The baseline survey included 445 households, with 333 households living in the rural and 112 in the urban area. During the first and second follow-up surveys, a total of 358 households (rural: 280; urban: 78) and 375 households (rural: 286; urban: 89) were interviewed. At baseline 15 of the sampled households could not be reached or were not willing to participate. The main reasons for drop-out in the follow-up surveys were households sampled at baseline relocated from the study area, the primary caregiver of the child had moved away due to domestic violence or was not available on the day of the interviews. The latter happened specifically during the first follow-up survey which coincided with the harvest season when many households were engaged in extensive farming and harvesting activities and therefore were not available for the interview.

Overall, the household size ranged from 2 to 21 persons with an average of six persons. Most of the households were headed by men (rural: 97.1%; urban: 98.9%). The ages of the women ranged from 17 to 75 years with a mean of 31.0 ± 9.20 years (two women were 17 years old and 14 women were older than 49 years), the older being grandmothers and aunts taking care of the young children. Whilst there were no significant differences in the mean height measurements between rural and urban women, the mean weight of women living in the urban area was significantly higher compared to those residing in the rural areas with a mean weight of 60.1 kg (SD *=* 11.90 kg) compared to 56.2 kg (SD *=* 11.10 kg) (*p* < 0.001). As a result, there was a significant difference in the mean body mass index (BMI) between women in rural and urban areas (22.27 kg/m^2^ vs. 24.18 kg/m^2^; *p* < 0.001) indicating differences in either dietary practices or lifestyle, such as lower physical activity.

The wealth index of households in the urban area was higher than in rural areas indicating that the households were wealthier compared to those in the rural settings (*p* < 0.05). A higher wealth index was also associated with a higher BMI. The prevalence of illiteracy was slightly higher among rural women compared to those living in the urban area (9% vs. 6.3%). Up to one quarter (27% vs. 26%) of women in both the rural and urban areas had completed secondary education with a significantly higher percentage of women (26%) from the urban area having an educational degree higher than secondary school compared to those from the rural area (12%) (*p* < 0.001). Further, the level of nutritional knowledge was significantly higher among women in the urban area compared to women living in the rural setting (*p* < 0.001).

Whilst more than half (62%) of the rural women were mainly involved in farming activities, only 28% of the women from the urban areas depended on farming. There, a significantly higher percentage of women generated their income from business activities (25%), wage employment (21.4%) and casual labour (17%) compared to women living in the rural settings (*p* < 0.001). Women from households in the rural areas had significantly more land for agricultural activities compared to those from the urban areas (*p* < 0.001). However, there were no significant differences in ownership of livestock and kitchen gardens among households from both the rural and urban settings. More women in the urban area (41.1%) had been worried (during the past 12 months) about becoming food insecure compared to women living in rural areas (34.8%), though these results were not significantly different.

The distances to the nearest health facilities, markets and water sources were significantly different between the urban and rural settings (*p* < 0.001). At least two-thirds of the women living in the rural area (65%) walked for at least 60 min to reach the nearest market, compared to 2.7% living in Kapchorwa TC, the urban area. Half of the women in the rural area (50%) and 8.9% of their counterparts from the urban areas used approximately 60 min or longer to reach the nearest health facilities. The proportion of women who spent 60 min or more to collect water was higher in the rural compared to the urban area (15% vs. 5%). 

### 3.2. Food Consumption Patterns of Women across Three Agricultural Seasons

**Figure 2:**
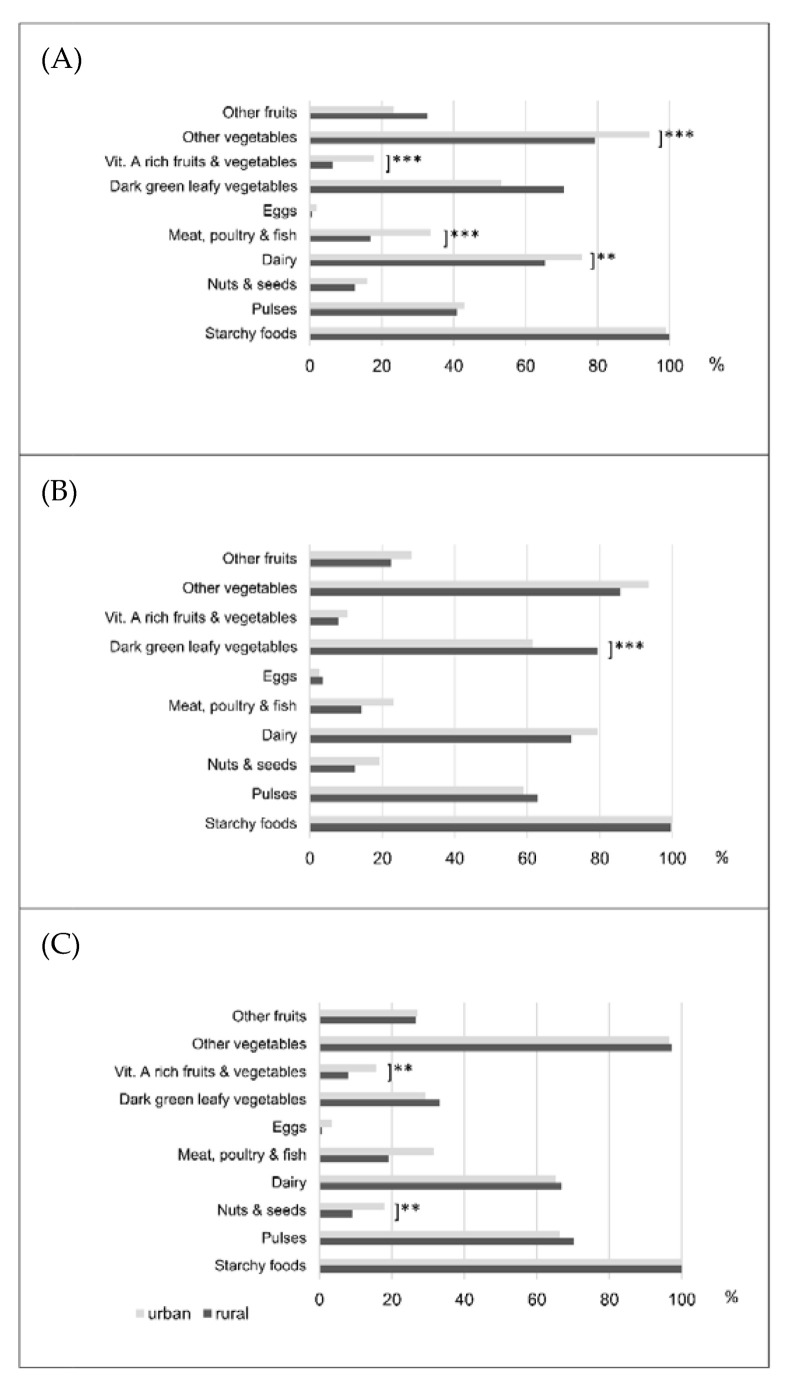
Food consumption pattern among women in rural and urban areas of the Kapchorwa District, Uganda, across agricultural seasons: (**A**) lean season: baseline in May/June 2016; (**B**) harvest season: 1st Follow-up in August 2016; (**C**) post-harvest season: 2nd Follow-up in January 2017; *p*-values statistically significant at ** *p* < 0.05 or *** *p* < 0.001 level.

The consumption of foods from different food groups by the women from the rural and urban settings across the three different seasons is presented in Figure 2. Nearly all households consumed foods from the food group ‘grains, white tubers and plantain’ in all three seasons. ‘Other vegetables’ were the second most consumed food ranging from nearly 80% to nearly 100% consumption during the lean and harvest season to 100% in both groups in the post-harvest period. ‘Dairy’ products were consumed at all times by more than 60% of the households with the highest consumption rates in the harvest seasons. ‘Dark green leafy vegetables’ were consumed by 50% and more women during lean and harvest season but reduced to less than 40% in the post-harvest season. The least consumed food groups were ‘eggs’, ‘nuts and seeds’, as well as pro-‘vitamin A-rich fruits and vegetables’. Similar findings were observed for these food groups during the other two seasons.

When comparing the food consumption patterns of women living in rural and urban settings, we found equal consumption rates of ‘pulses’ during the lean season. Women in the urban area consumed significantly more animal source foods, such as ‘meat, poultry and fish’ (*p* < 0.001), ‘dairy products’ (*p* < 0.05), as well as foods from the food groups ‘vitamin A-rich fruits and vegetables’ and ‘other vegetables’ (both *p* < 0.001). By contrast, women in rural settings consumed significantly more ‘dark green leafy vegetables’ during the lean and harvest season compared to women in the urban area (70.6% vs. 53.3% and 79.3% vs. 61.5%, respectively). Women in the urban setting consumed significantly more foods from the food groups ‘nuts and seeds’ (18%; *p* < 0.05) as well as ‘vitamin A-rich fruits and vegetables’ (15.7%; *p* < 0.05) during the post-harvest season.

The differences in food consumption patterns between women living in rural and urban areas during the different seasons are summarised in Table 2. When comparing the food consumption patterns of women from rural areas between the harvest and lean season, significant changes among different food groups were observed. During the harvest season, rural women consumed significantly more ‘pulses’, ‘eggs’, ‘dark green leafy vegetables’ and ‘other vegetables’. On the other hand, significantly fewer foods from the food groups ‘grains, white tubers and plantain’ as well as ‘other fruits’ were consumed. The number of food groups consumed by these women during both agricultural seasons showed a significantly higher consumption during the harvest season (mean: 4.60 food groups) compared to the lean season (mean: 4.25 food groups; (*p* < 0.001)). Similar results were obtained for the food groups ‘pulses’ and ‘other vegetables’ when comparing the post-harvest and harvest seasons. A highly significant decline was found regarding the consumption of ‘dark green leafy vegetable’ (−46.1%; *p* < 0.001), whilst fewer ‘eggs’ were consumed by 2.9% of rural women (*p* < 0.05).

A significant decline in the consumption of ‘vitamin A-rich fruits and vegetables’ by 7.5% (*p* < 0.05) was thus discovered when comparing the harvest and the lean season among women in the urban area. Significant differences between the third and second agricultural seasons were found for the food groups ‘dairy’ and ‘dark green leafy vegetables’. The consumption of the latter food group also differed significantly when comparing the post-harvest with the lean season. In total, the consumption of ‘dark green leafy vegetables’ dropped by 24.1%.

### 3.3. Dietary Diversity of Women across Agricultural Seasons

Overall, the mean dietary diversity score for women (WDDS) at the lean, harvest and post-harvest season was 4.33 ± 1.21, 4.63 ± 1.30 and 4.36 ± 1.21, respectively. The mean number of food groups covered in the diet of rural women dropped significantly from 4.60 to 4.31 food groups (*p* < 0.01) between harvest and post-harvest season. No significant difference was observed between the post-harvest and lean season (*p* = 0.317), though significant changes were obtained for ‘pulses’, ‘dark green leafy vegetables’ and ‘other vegetables’. This was different for the women living in the urban area, whose mean number of food groups did not differ significantly between the three seasons.

**Figure 3:**
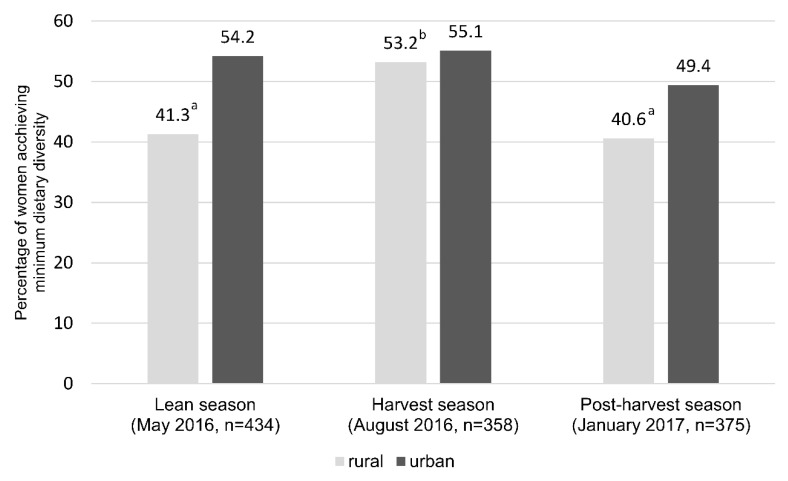
Percentage of women achieving minimum dietary diversity in rural and urban settings at the different agricultural seasons. Wilcoxon Test, a/b = statistically significant different at *p* < 0.001 level.

In the lean season, 41.3% of the women in rural and 54.2% in urban settings achieved the minimum dietary diversity (MDD-W) by consuming foods from at least five or more food groups (out of 10). The proportion of women who achieved MDD-W increased during the harvest season by 12% among the rural and by 1% in the urban group. However, only 40.6% of the women from the rural and 49.4% in the urban areas achieved MDD-W during the post-harvest season (*p* < 0.05; Figure 3).

### 3.4. Relationship between Women Dietary Diversity and Household’s Wealth Index

The two-way ANOVA was performed in order to test for relationships between dietary diversity scores of the women across the agricultural seasons and the wealth of the household. The results estimated that there was no significant relationship between MDD-W and wealth. However, a trend was observed with food consumption patterns among poorer households in rural areas being more affected by season, particularly in the lean and post-harvest season (*p* < 0.05).

In addition, Spearman’s rho correlation test was conducted to evaluate possible correlations between dietary diversity and wealth across agricultural seasons. The results support our findings of the two-way ANOVA (Table 3). Low, but significant positive correlations were found for women living in rural settings and the wealth of the household and the lean season (*p* < 0.01), as well as for the post-harvest season (*p* < 0.001). In the urban area, seasonality was not correlated with the wealth index and therefore did not play any significant role with regard to women’s dietary diversity.

The linear mixed model showed that women in rural areas had a significantly lower mean dietary diversity score than women in the urban area which was not influenced by season (estimated marginal means (EMM) = 0.33 (*p* < 0.015)) (Table 4).

Further analysis confirmed the observed association between rural women’s dietary diversity score and wealth, i.e., dietary diversity rose significantly (*p* < 0.001) by 0.8 units if the wealth index rose by 10 units for rural but not for urban women. Rural women with a lower wealth index of −2.4 have a higher risk of having a low dietary diversity compared to urban women (rural–urban difference = −0.44 WDDS units; *p* = 0.004) and compared to rural women with a medium (0) or high wealth index (2.4) (rural–urban difference = −0.22 (*p* = 0.04) and <0.00002 units (*p* = 0.99, respectively). The association between dietary diversity, rural and wealth changed after the inclusion of educational level into the model, indicating that formal education may be a mediator for dietary diversity which outlays the effect of living in a rural area

## 4. Discussion

The findings of the present study point to divergent seasonal trends in dietary diversity among women in rural and urban settings across agricultural seasons in the Kapchorwa District, Uganda. The variation in dietary diversity was more pronounced among women in rural settings, who mainly depend on income from agricultural activity. Although women in rural areas had significantly more arable land available, dietary diversity was lower during all seasons compared to women living in the urban area. Hence, placing emphasis exclusively on agricultural activities appears to be insufficient to achieve adequate dietary diversity throughout the year.

The proportion of women achieving minimum dietary diversity among rural women (MDD-W) was 12% higher during the harvest season compared to the lean season, whilst the change was very low (1% distinction) for women living in the urban area. In both sites, rural and urban, MDD-W was lower in the post-harvest season compared to the lean season. Similar findings were reported in studies from Burkina Faso and Benin [3,43]. Higher dietary diversity during the lean season might occur through higher food expenditure from a household’s income and the utilisation of other food sources from the wild or from gifts in both settings. Collection of foods from the forest was more likely adopted by women living in rural settings and close to the forest [44]. This was also found in our region when women used the collected foods to supplement their diets during the lean season. This finding explains the significant difference in the consumption of dark green leafy vegetables between seasons, where they are commonly freely available in the Mt. Elgon forest around the lean season [44]. The availability of dark green leafy vegetables on the urban markets is limited throughout the year. In times of scarcity, they were imported from larger markets in neighbouring cities.

During the post-harvest season the highest consumption of animal source foods, such as ‘meat, poultry and fish’, was reported by the rural women. In the Ugandan setting, meat consumption is very common during circumcisions season regularly carried out in December. At Christmas and New Year, animal source food items are also commonly consumed as well as presented as gifts to family members [45,46]. Whilst chicken meat is more affordable, it is not surprising that the consumption of eggs was much lower at that time of the year. By contrast, urban women had the highest egg consumption during the post-harvest season. They generated significantly more income through off-farm activities compared to women in rural areas. Among women in the urban area of the Kapchorwa District, cash income can be used for food purchases at the market enabling them to substitute less available and accessible foods during the lean season. They can thus maintain some dietary diversity.

In Uganda in general, urban households have better access to a variety of foods and a higher purchasing power to buy more diverse and nutritious food items compared to households located in remote areas [29]. In this study, we observed that the dietary diversity of “wealthier” rural women was not different from that of wealthier urban women in the same wealth category which indicates similar access to food for both groups. Food purchases from the market might have supplemented the own but lower food production, especially in the lean season. Positive associations between off-farm activities and dietary diversity during the lean season in sub-Saharan African countries have been also reported by Fraval and colleagues [47]. However, unconstrained access to income from non-farm activities may improve households’ income and access to foods with a negative impact on agricultural production and land conservation [48] and, depending on the food choices, increased the risk for a double burden of malnutrition [49].

Price fluctuations (may) occur in line with demand and availability of commodities at markets. Cereals are more prone to such volatility than other goods which do not depend on seasonality and are not considered as staple foods, such as animal source foods, such as eggs, milk and meat. Those price fluctuations can have a negative impact on dietary intake and can cause nutrient deficiencies [50,51,52], when nutritious foods are substituted by staple foods that are usually the cheapest source of dietary energy and which “fill the stomach”, such as cereals and starchy roots [53]. In the study region, posho, made of maize, is the basic component of each meal, is highly appreciated and culturally accepted. It is the least likely food to be skipped from the plate in terms of food shortage. This explains why there were very limited differences across the seasons in the consumption of foods from the food group “grains, white tubers and plantain”. Gilbert and colleagues examined the extent of seasonal food availability and its effect on the food prices of several items across seven Sub-Saharan African countries. They reported that fruits and vegetables were affected most by price volatility which, in turn, depended on seasonal availability [54].

In developing countries, poor households and small-holder farmers spend up to 80% of their monetary income on food [53]. In the present study, the wealth status of the households was significantly higher among urban women. Compared to their rural counterparts, these women in the urban region had higher financial resources and consumed more foodstuffs from the food groups ‘vitamin A-rich fruits and vegetables’ and ‘other vegetables’. Both foods were available on the markets during the lean season.

Access to food markets has been promoted as an important strategy to increase dietary diversity among all family members in developing countries [55]. Market access seems to play an even more dominant role than farm production diversity when it comes to food diversity among individuals [56,57]. Especially for the supply of fresh foodstuffs, such as fruits, vegetables and dairy products, market access is a great advantage [56]. In the present study, urban women had significantly better access to markets, i.e., needed less time to reach formal and informal marketplaces to purchase food, which might facilitate their diverse food consumption patterns. At the same time, it may explain their lower intake of dark green leafy vegetables for which market availability was limited.

Nevertheless, the area of land available for agricultural production cannot be disregarded. Especially in rural regions with limited market access, subsistence cropping helps to prevent food insecurity and increase dietary diversity. More poor households benefit from producing their own food, which enables them to avoid high expenditure on food, especially when prices are rising [58]. Chakona and Shackleton reported a positive relationship between subsistence agricultural production and women’s dietary diversity in South Africa [59]. In addition, Zanello and colleagues found a positive relationship between crop diversity and dietary diversity in Afghanistan, except for the lean season [60]. In the Kapchorwa District, women in rural settings had significantly more access to land for crop production than women living in urban areas. Given the majority of small-holder farmers in the Kapchorwa District do not cultivate more than four food crops, the promotion of cropping diversity may help to increase dietary diversity. However, this alone would not be sufficient, because crop diversity is always lower during the lean season.

It is, therefore, important to place emphasis on improved methods of food storage and preservation (e.g., improved storage for beans, preserve vegetables through drying, watering of fresh leafy vegetables to reduce post-harvest loss during marketing) to achieve and maintain dietary diversity during the lean season. Positive correlations between storage facilities and dietary diversity have been reported in other studies [61,62]. Tesfaye and Tirivayi also observed that storage facilities became more important with increasing distance from markets [62]. Therefore, storage and conservation techniques can help to improve and maintain dietary diversity throughout the year, particularly in remote areas. This would increase the flexibility of rural households to exchange more food groups across seasons, which seems to be currently only feasible for the food groups ‘dark green leafy vegetables’ with ‘other vegetables’ and vice versa. In comparison, urban households have greater possibilities of replacing currently unavailable foods with available foods, and so better maintain dietary diversity across seasons.

We observed a positive association between the wealth of the household and food consumption pattern during the lean and post-harvest season in the rural setting, whilst wealth did not play a significant role in the consumption pattern in the urban group. The regression model confirmed that the extremely poor in the rural area were more affected by seasonality than the less poor rural women, which is not surprising. Several studies have revealed a positive effect of wealth on market access and dietary diversity, but we also assume more market activities among the urban group in general, poor and wealthy, as their main occupation was less likely to be agriculture. They had thus to rely more on the food available on the markets. The distance to the market for rural women was significantly longer compared to the urban environment and thus transport costs (time or financially) further reduced the ability of rural women to purchase food on the market. These costs may be reduced by improving the road infrastructure which, in turn, will improve the public transport system as villages are accessible more easily.

Women’s education functioned as a mediator for improved dietary diversity as could be seen in the regression model adjusted for educational level: The difference in rural and urban dietary diversity reduced after adjusting for women’s educational level. However, the overall seasonal effect between lean and harvest season remained. Considering also the commonly lower nutritional knowledge among the rural compared to the urban women, we recommend linking participation in markets with agricultural activities, such as extension for crop diversification and conservation agriculture, and nutritional knowledge, to improve and maintain dietary diversity in overcoming seasonal variations, especially among rural households.

### Strengths and Limitations of the Study

Dietary diversity scores were calculated using an open 24-h-recall template and assessed by a well-trained team of enumerators following the measurement guidelines for MDD-W [13]. The correct placement of the consumed foods was done by a team of experts which minimized the risk for misclassification of the foods. No differentiation was made between weekdays and the weekend but feast days were intentionally not included. This may have resulted in estimation bias considering that on religious days, such as Fridays or Sundays, special foods may have been served alongside prayers or services. Seasonality was described using three time points only, i.e., three 24-h-recalls. This way, less well-known seasons which may potentially influence food intake may have been overlooked.

Kapchorwa TC is officially a district town area with a few food stores, small supermarkets and public and informal market stands, where various foods can be purchased. The town has one main road which basically ends at the forest as a “dead-end”. This definition of “urban” might exaggerate the situation of the population in Kapchorwa TC compared to other cities. Due to the low agrobiodiversity in the Kapchorwa District, the diversity of food offered on the Kapchorwa markets is usually limited and people have to leave the district to purchase vegetables and fruits. Access to vegetables and fruits by the women in Kapchorwa TC might thus not be comparable with women in larger cities if other factors remain the same.

The range of foods offered was limited at the time of this study due to a drought which negatively impacted the harvest, water and food availability [24,34]. Consequently, the dietary diversity level of the women in this study might have been underestimated.

The performance of a joined wealth index for urban and rural populations has been discussed in various studies [39,40]. We decided to use a composite set of variables to estimate the wealth index including a set of farming tools and land size which may be biased towards the rural poor.

## 5. Conclusions

Our findings provide evidence for fluctuations in women’s dietary diversity across agricultural seasons and village locations. There is not necessarily the need to strengthen farmers’ off-farm activities, but to improve the income from agricultural activities to close seasonal gaps because wealth and seasonal dietary diversity was linked especially among the rural farm households. Nutrition education to improve nutritional knowledge and raise awareness among the less educated women linked with agricultural extension services aimed at improving vegetable and fruit availability for dietary diversity is strongly recommended. Diversifying cropping patterns may help to improve accessibility to the more perishable foods on-farm but may also be an option to strengthen the availability of vegetables for the urban population who depend more on markets compared to their counterparts in the more remote areas. Further research is thus needed to determine which nutrition-sensitive agricultural innovations will enhance crop diversity for farmers’ own consumption and market transactions, and in combination with improved preservation methods, might help to increase and maintain high dietary diversity across all seasons among women in both, rural and urban areas.

## Figures and Tables

**Table 1:** Households’ main characteristics stratified by rural and urban areas at baseline (May 2016).

	All (*n =* 445)		Rural (*n =* 333)		Urban (*n* = 112)		Rural vs. Urban
	%	n	%	n	%	n	*p-*Value
**Household head**							0.337 ^§^
Male	97.5	385	97.1	297	98.9	88	
Female	2.5	10	2.9	9	1.1	1	
**Occupation of household head**							<0.001 ^§^
Crop farming	53.0	236	61.6	205	27.7	31	
Business	13.1	59	9.3	31	25.0	28	
Casual labour	10.1	45	7.8	26	17.0	19	
Wage employment	17.1	76	15.6	52	21.4	24	
Others	6.7	29	5.7	19	9.0	10	
**Land size**							<0.001 ^§^
0.1–1.24 acre	44.8	199	40.2	134	58	65	
1.25–2.9 acre	35.8	159	38.1	127	28.6	32	
>3.0 acre	19.4	86	21.6	72	12.5	14	
**Distance to nearest…**							
Health facility ≥ 1 h	37.3	166	46.8	156	8.9	10	<0.001 ^§^
Market ≥ 1 h	49.9	122	65.7	219	2.7	3	<0.001 ^§^
Water source ≥ 1 h	12.6	56	14.8	49	5.4	6	<0.001 ^§^
**Marital status of the women**							0.925 ^§^
Married–monogamous	75.5	336	75.1	250	76.7	86	
Married–polygamous	18.0	80	19.5	65	13.4	1	
**Women’s level of school education**							<0.001 ^§^
Illiteracy level	9.2	38	9.3	31	6.3	7	
Primary completed (7 years)	39.1	174	42.9	143	27.7	31	
Secondary completed (11 years)	26.5	118	26.7	89	25.9	29	
Higher than secondary (12–17 years)	15.5	69	12.0	40	25.9	29	
**BMI categories (women)**							<0.001 ^†^
Underweight ≤18.49 kg/m^2^	11.0	44	12.5	37	6.3	7	
Normal weight 18.50–24.99 kg/m^2^	63.9	255	67.9	201	52.4	54	
Overweight ≥ 25.00 kg/m^2^	25.1	199	19.6	58	40.8	42	
	**Mean (SD)**	**n**	**Mean (SD)**	**n**	**Mean (SD)**	**n**	
**Household size (number)**	6.53 (2.67)	445	6.49 (2.53)	333	6.67 (3.07)	112	0.95 ^†^
**Wealth Index (WI)**	0.001 (2.40)	89	−0.30 (1.98)	333	0.90 (3.19)	112	<0.05 ^†^
Lowest wealth level (1. Quintile of WI)	−2.84 (0.86)	89	−2.91 (0.85)	69	−2.61 (0.89)	17	
Less poor	−1.22 (0.30)	89	−1.21 (0.29)	72	−1.26 (0.35)	17	
Moderate wealthy	−2.54 (0.25)	89	−0.26 (0.26)	69	−0.23 (0.22)	20	
Nearly wealthy	0.76 (0.39)	89	0.75 (0.26)	70	0.83 (0.39)	19	
Highest wealth level (5. Quintile of WI)	3.56 (2.20)	89	2.89 (1.29)	53	4.55 (2.84)	36	
**Women’s age (years)**	30.95 (9.22)	443	31.0 (9.30)	331	30.9 (9.00)	112	0.903 ^†^
Women’s weight (kg) *	57.4 (11.46)	399	56.2 (11.12)	296	60.1 (11.90)	103	<0.001 ^†^
Women’s height (cm)	158.89 (5.93)	408	158.87 (5.87)	303	158.94 (6.13)	105	0.947 ^#^
BMI (kg/m^2^)	22.76 (4.36)	399	22.3 (4.18)	296	24.2 (4.56)	103	<0.001 ^†^

† Mann–Whitney test; # independent *t*-Test, § Pearson chi-square test excluding pregnant women; WI *=* wealth index (min-max *=* −5.40–11.58); * includes weight of minimum clothing; BMI *=* Body Mass Index.

**Table 2:** Changes in proportions of women (%) eating foods from the ten food groups counted in the women’s dietary diversity scores over time in rural and urban areas in the Kapchorwa District, Uganda.

	Harvest versus Lean Season			Post-Harvest versus Harvest Season			Post-Harvest versus Lean Season		
10 Food Groups of MDD-W ^a^	Rural	Urban	*p*-Value	Rural	Urban	*p*-Value	Rural	Urban	*p*-Value
Grains, white tubers and plantain	−0.4	+0.9		+0.4	0.0		0.0	+0.9	
Pulses	+21.9	+16.0	<0.001	+7.4	+7.3	<0.05	+29.3	+23.3	<0.001
Nuts and seeds	0.0	+3.3		−3.4	−1.2		−3.4	+2.1	
Dairy	+6.7	+3.8		−5.3	−14.3	<0.05	+1.4	−10.5	
Meat, poultry and fish	−2.5	−10.5		+4.9	+8.4		+2.4	−2.1	
Eggs	+3.0	+0.7	<0.05	−2.9	+0.8	<0.05	+0.1	+1.5	<0.05
Dark green leafy vegetables	+8.7	+8.2	<0.05	−46.1	−32.3	<0.001	−37.4	−24.1	<0.001
Vit. A rich fruits and vegetables	+1.5	−7.5	<0.05	+0.1	+5.4		+1.6	−2.1	
Other vegetables	+6.5	−0.8	<0.05	+11.5	+3.0		+18.0	+2.2	<0.001
Other fruits	−10.2	+4.8	<0.05	+4.1	−1.2		−6.1	+3.6	

^a^ FAO and FHI 360 (2016): Minimum Dietary Diversity for Women—A Guide to Measurement. *p*-values (only shown when significant) indicate differences in food consumption pattern among women across agricultural seasons within food groups from the Wilcoxon test; green shaded numbers indicate a significant increase and red a significant decrease. Lean season: baseline in May/June 2016; harvest season: 1st Follow-up in August 2016; post-harvest season: 2nd Follow-up in January 2017.

**Table 3:** Spearman correlations between women’s dietary diversity and household wealth index across agricultural seasons.

Household Location	Women Dietary Diversity at Seasons ^#^	Household Wealth Index (Spearman’s Rho Coefficient)
rural (n *=* 243)	Lean	0.163 **
	Harvest	0.051
	Post-harvest	0.253 ***
urban (n *=* 63)	Lean	0.170
	Harvest	−0.123
	Post-harvest	−0.106

^#^ Lean season, baseline, May 2016; Harvest season, 1st follow-up, August 2016; Post-harvest season, 2nd follow-up, January 2017; ** = *p* < 0.05 and *** = *p* < 0.001.

**Table 4:** Estimated marginal means with 95% CI women’s dietary diversity scores across three different seasons adjusted for wealth (Model 1) and wealth and educational level (Model 3).

		Model 1 (Unadjusted)			Model 2 (Adjusted for Wealth)			Model 3 (Adjusted for Wealth and Women’s Educational Level)		
Season *	Living Area	Estimated Marginal Mean	95% Confidence Interval		Estimated Marginal Mean	95% Confidence Interval		Estimated Marginal Mean	95% Confidence Interval	
Lean	Rural	4.3	4.1	4.4	4.3	4.1	4.4	4.3	4.2	4.5
	Urban	4.6	4.4	4.8	4.6	4.4	4.8	4.6	4.3	4.8
Harvest	Rural	4.6	4.4	4.7	4.6	4.5	4.8	4.7	4.5	4.8
	Urban	4.8	4.5	5.0	4.8	4.5	5.0	4.7	4.5	5.0
Post-harvest	Rural	4.3	4.2	4.5	4.3	4.2	4.5	4.4	4.3	4.5
	Urban	4.5	4.3	4.8	4.5	4.3	4.8	4.5	4.3	4.8

* Lean season, baseline, May 2016; Harvest season, 1st follow-up, August 2016; Post-harvest season, 2nd follow-up, January 2017.

## Data Availability

The raw data supporting the conclusions of this article will be made available by the authors, without undue reservation. The data are not publicly available due to the privacy restrictions of the farm families.
